# *Cryptosporidium parvum virus 1* genome sequences from pediatric diarrhea cases in coastal Kenya

**DOI:** 10.1128/mra.00349-25

**Published:** 2025-09-04

**Authors:** Charles N. Agoti, Regina Njeru, Mike Mwanga, Martin Mutunga, Sanyam Paresh Shah, Ting-Yu Chu, D. James Nokes, Matthew Cotten, My V. T. Phan

**Affiliations:** 1 Wellcome Trust Research Programme (KWTRP), Kenya Medical Research Institute (KEMRI) 118982, Kilifi, Kenya; 2 Pwani University 270495, Kilifi, Kenya; 3 International Livestock Research Institute (ILRI) 54661, Nairobi, Kenya; 4 Institute for Infectious Disease, University of Bern 27210, Bern, Switzerland; 5 College of Health Solutions, Arizona State University 7864, Phoenix, Arizona, USA; 6 Complex Adaptive System Initiative, Arizona State University 7864, Scottsdale, Arizona, USA; Katholieke Universiteit Leuven, Leuven, Belgium

**Keywords:** *Cryptosporidium parvum*, enteric, diarrhea, Kenya, virus genomics

## Abstract

We report nine nearly complete *Cryptosporidium parvum virus 1* genome sequences (both segments) recovered from stool samples of pediatric diarrhea patients admitted to Kilifi County Hospital, coastal Kenya. This will be an important resource for monitoring *C. parvum* infections in humans.

## ANNOUNCEMENT

*Cryptosporidium parvum virus 1* (CSpV1) belongs to genus *Cryspovirus* in the *Partidiviridae* family of viruses (1), with a two-segment double-stranded RNA genome, encoding RNA polymerase (dsRNA1) and capsid protein (dsRNA2). CSpV1 is thought to be a symbiont and carried by all *Cryptosporidium* parasites and passed on by vertical inheritance (2) with no strong evidence for extracellular infectious forms of the virus. CSpV1 is reported to upregulate type-1 interferon responses promoting *Cryptosporidium* infection (3). CSpV1 is of interest as a target for *Cryptosporidium* diagnosis, for tracking *Cryptosporidium* infections in the community and for studying the biology of modulating host immune responses to *Cryptosporidium* spp. Here, we report nine nearly complete genomes (both segments) generated from pediatric diarrhea patient samples admitted to a county referral hospital in coastal Kenya in 2012–2014. Previous work had found *Cryptosporidium* in ca. 10% of childhood diarrhea cases in this cohort (4). These genome sequences of CSpV1 from human *C. parvum* infections may be useful in studies of *Cryptosporidium* and CSpV1 biology.

As part of the longitudinal rotavirus surveillance to document infections associated with pediatric diarrhea, stool samples were obtained from children hospitalized for diarrhea in the Kilifi County Referral Hospital (KCRH). Consent was obtained from the children’s parents or legal guardians, and ethical approvals for the study protocol were obtained from SERU (Scientific and Ethics Review Committee; SERU #3624). Total nucleic acid was extracted from stool samples using the Boom method (5), followed by reverse transcription using random non-ribosomal primers (6) and second-strand DNA synthesis using 5U Klenow fragment (New England Biolabs), as described previously (7). The resulting DNA was sheared to 400–500 nt (Covaris instrument), followed by library preparation using NEBNext end repair and Kapa HiFi kits and sequenced on Illumina HiSeq 2500, generating 140,000 to 40 million 250 nt paired-end reads per sample. After removing low-quality reads using QUASR (8) (median Phred > 35, read length ≥ 175 nt), *de novo* assembly was performed using SPades (9) and *Cryspovirus* contigs were identified using kmer matching to a *Cryspovirus* database (all GenBank sequences with taxon id 675059) with MMseqs2 (10). Quality of assembled contigs and coverage were checked by mapping reads to either NC_038843 (RNA1, segment size 1,836 bp) or NC_038844 (RNA2, segment size 1510 bp) reference sequences using Minimap2 (11). Default parameters were used except where otherwise noted. A summary of the samples, metrics, and resulting genome segment sequences is found in Table 1.

**TABLE 1:** Sample details, read metrics, and accession links

Sample ID	Collection date	Segment length (nt)	GC content (%)	Total quality-controlled reads	Total CSpV1-mapped reads	SRA accession number read lane1	SRA accession number read lane2	CSpV1 GenBank accession number
20718_23_RNA1	17-May-12	1,767	39.96	166,317	6,546	ERR2217790	ERR2217885	PV275245
20718_23_RNA2	17-May-12	1,488	40.41	166,317	8,464	ERR2217790	ERR2217885	PV275254
20718_50_RNA1	23-Nov-12	1,829	38.28	41,491,316	1,082	ERR2217817	ERR2217912	PV275246
20718_50_RNA2	23-Nov-12	1,513	40.71	41,491,316	829	ERR2217817	ERR2217912	PV275255
20718_84_RNA1	13-Jul-13	1,788	37.75	943,558	2,057	ERR2217851	ERR2217946	PV275247
20718_84_RNA2	13-Jul-13	1,466	40.3	943,558	1,097	ERR2217851	ERR2217946	PV275256
20719_15_RNA1	1-Nov-13	1,799	37.42	414,688	30,904	ERR3178976	ERR3179071	PV275248
20719_15_RNA2	1-Nov-13	1,469	40.44	414,688	116,452	ERR3178976	ERR3179071	PV275257
20719_1_RNA1	6-Dec-13	1,720	37.42	746,693	30,877	ERR3178962	ERR3179057	PV275249
20719_1_RNA2	6-Dec-13	1,380	40.63	746,693	116,376	ERR3178962	ERR3179057	PV275258
20719_21_RNA1	26-Aug-13	1,836	38.46	5,139,618	251	ERR3178982	ERR3179077	PV275250
20719_21_RNA2	26-Aug-13	1,501	40.41	5,139,618	565	ERR3178982	ERR3179077	PV275259
20719_56_RNA1	21-Feb-14	1,826	37.8	20,058,833	2,378	ERR3179017	ERR3179112	PV275252
20719_56_RNA2	21-Feb-14	1,513	40.91	20,058,833	1,847	ERR3179017	ERR3179112	PV275261
20719_8_RNA1	24-Jun-13	1,704	37.63	150,285	551	ERR3178969	ERR3179064	PV275253
20719_8_RNA2	24-Jun-13	1,221	39.37	150,285	487	ERR3178969	ERR3179064	PV275262
20719_26_RNA1	31-Aug-13	1,730	38.34	1,466,249	5,266	ERR3178987	ERR3179082	PV275251
20719_26_RNA2	31-Aug-13	1,493	40.65	1,466,249	3,808	ERR3178987	ERR3179082	PV275260

**Fig 1:**
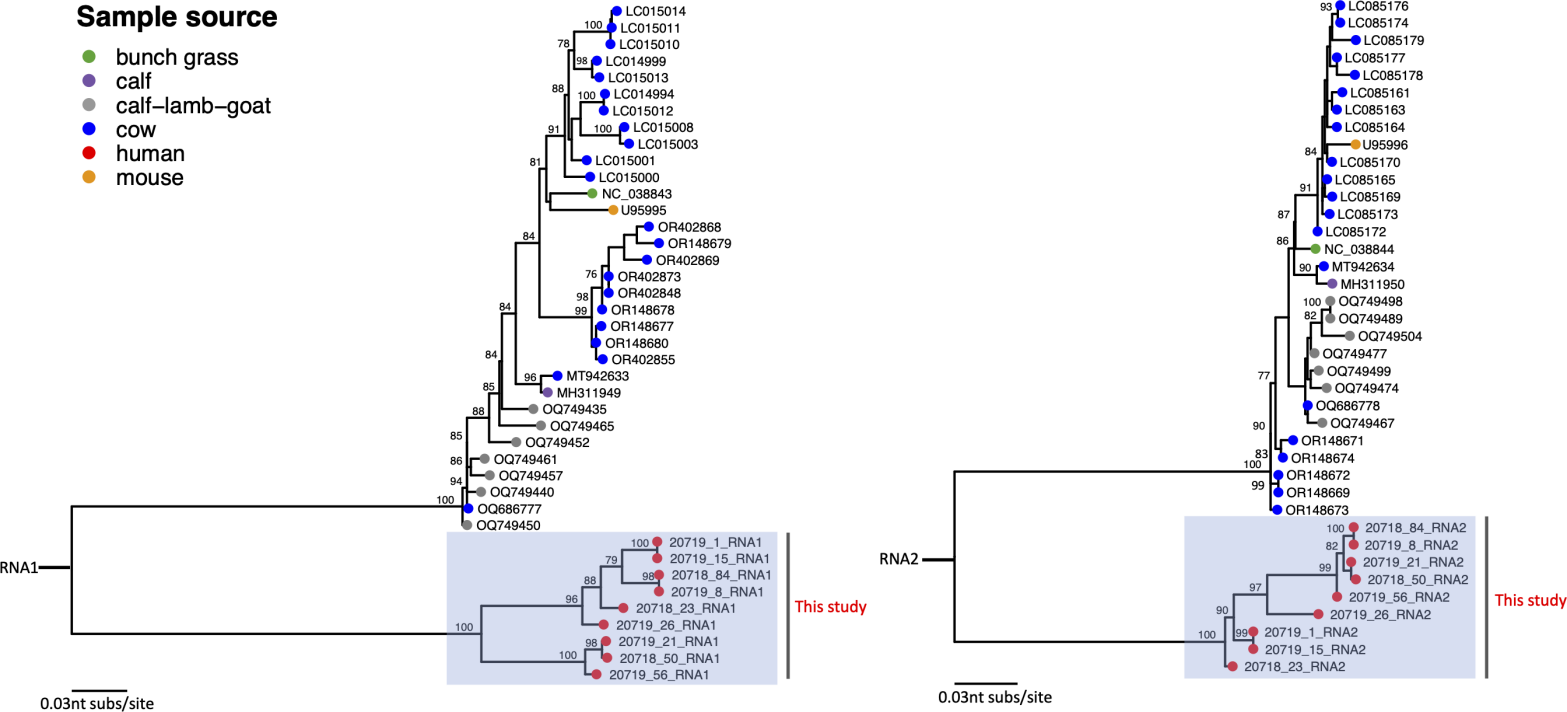
Phylogenetic trees comparing the human CSpV1 virus complete coding sequences from Kenya with other available genomes from GenBank (accession numbers listed for each sequence used). Trees were mid-pointed rooted. Nodes were colored by sample source, human origin sequences from this study are indicated with red nodes in the gray boxes, bootstrap values (>75%) indicate at branch points. Left panel: dsRNA1 genome segment; Right panel: dsRNA2 genome segment.

Global CSpV1 comparative genomics suggest sufficient sequence diversity for use in tracing *Cryptosporidium*. We compared the reported sequences with publicly available CSpV1 dsRNA1 and dsRNA2 sequences. Reference sequences from GenBank were aligned with the reported CSpV1 using MAFFT v7 (12). Phylogenetic trees were constructed in RaxML-NG (13) under the GTR-G-I4 model of evolution with 100 bootstraps. The reported CSpV1 sequences were closely related to each other and distinct from CSpV1 sequences from other *Cryptosporidium* sources from other regions (Fig. 1). Given the challenges in detecting *Cryptosporidium* parasites, the rapid detection of CSpV1 virus (e.g., real-time PCR) could provide an alternative indirect diagnosis and monitoring of *Cryptosporidium* parasites.

## Data Availability

The genome sequences described in this report have been deposited in GenBank under accession numbers PV275245–PV275262, with individual hyperlink for each virus segment listed in Table 1. The raw sequencing data are available in the NCBI SRA archive (BioProject accession number PRJEB6505), with corresponding accession numbers and hyperlinks for each sample listed in Table 1.
